# Flow restoration during mechanical thrombectomy for large vessel occlusion is associated with an immediate reduction of systemic blood pressure

**DOI:** 10.1177/23969873241274512

**Published:** 2024-08-30

**Authors:** Anna Andriana Kyselyova, Caspar Brekenfeld, Lucas Meyer, Helena Guerreiro, Gabriel Broocks, Susan Klapproth, Tobias Faizy, Christian Heitkamp, Malte Issleib, Jens Fiehler, Fabian Flottmann

**Affiliations:** 1 Department of Diagnostic and Interventional Neuroradiology, University Medical Center Hamburg-Eppendorf, Hamburg, Germany; 2 Clinic of Neuroradiology, University Hospital Zurich, Zurich, Switzerland; 3 Department of Anesthesiology, University Medical Center Hamburg-Eppendorf, Hamburg, Germany

**Keywords:** Stroke, thrombectomy, systolic blood pressure

## Abstract

**Introduction:**

Managing blood pressure in patients with large vessel occlusion affects infarct size and clinical outcomes. We examined how restoring blood flow impacts systemic blood pressure during mechanical thrombectomy.

**Patients and Methods:**

Patients with large vessel occlusion in the anterior circulation undergoing mechanical thrombectomy between June 2016 and January 2018 were screened. We included those treated under local anesthesia or conscious sedation and analyzed standardized anesthesia protocols to assess systolic and diastolic blood pressure levels throughout the procedure. The primary outcome was the change of blood pressure, compared 5 min before versus 5 min after the last recanalization attempt. Successful reperfusion was defined as Thrombolysis in Cerebral Infarction score ⩾ 2b.

**Results:**

Of 134 patients, 117 (87%) achieved successful angiographic reperfusion, showing a notable systolic blood pressure drop 5 min after flow restoration (10.2 ± 14.6 vs 3.24 ± 8.65 mm Hg, *p* = 0.009). Successful angiographic reperfusion was a significant predictor for this decrease in multivariable logistic regression: OR = 1.34 (95% CI: 1.03–1.73, *p* = 0.0299). Among 66 patients not given circulation-affecting meds, a significant systolic pressure reduction was also observed (155 ± 17 mm Hg to 148 ± 17 mm Hg ; *p* < 0.001). No diastolic pressure changes were significant.

**Discussion and Conclusions:**

Flow restoration was associated with an immediate reduction of systolic blood pressure values in patients undergoing mechanical recanalization under local anesthesia or conscious sedation. This suggests a complex interplay between endovascular stroke therapy and cardiovascular hemodynamics.

## Graphical abstract

**Graphical abstract:**
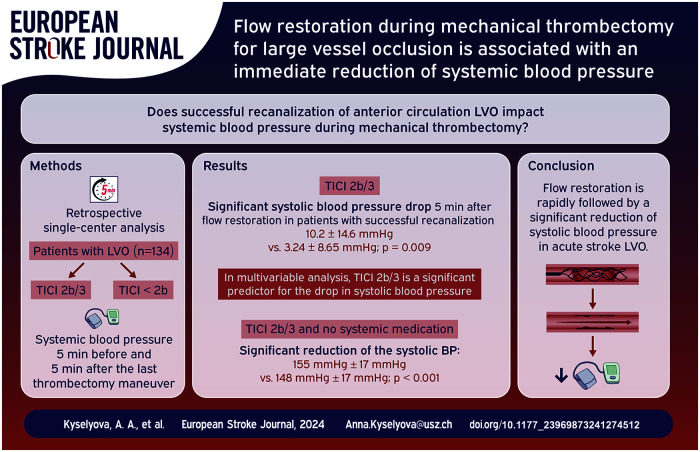

## Introduction

The introduction of mechanical thrombectomy (MT) has had a significant influence on treatment strategies for patients with acute ischemic stroke and their potential clinical outcomes. Presently, MT has emerged as the established method for treatment of large and medium vessel occlusions (LVO, MeVO).^1–7^ Nevertheless, a substantial proportion of patients exhibits poor clinical outcome despite successful reperfusion.^8^ Hence, there is still a significant requirement for additional approaches to improve the outcomes of patients undergoing MT. One of these parameters is the control of the systemic blood pressure before, during and after the endovascular treatment. Prognostic factors for neurological outcomes after an acute ischemic stroke caused by large-vessel occlusion and treated by MT predominantly center on the expansion of the infarct core. The rate of ischemic core expansion is inter-individually variable and primarily depends on the efficacy of collateral circulation, a factor susceptible to modification by cerebral hemodynamic conditions.^9,10^ Additionally, intraprocedural hypotension has also been shown to be an independent predictor for poor neurological outcome in patients with acute ischemic stroke and treatment under general anesthesia, which could be explained by post induction hypotension and effects of anesthetic drugs resulting in worsening of the cerebral ischemia.^11–14^ The recently published INTERACT 4 study has shown that reducing blood pressure prior to hospital admission was correlated with a decreased likelihood of poor functional outcome in hemorrhagic stroke patients. Conversely, in patients with cerebral ischemia, a higher risk of poor functional outcomes was associated with pre-hospital blood pressure reduction.^15^ Carvalho Dias et al. have shown that the spontaneous systolic blood pressure drop within the first hour after mechanical thrombectomy is an early predictor of significant neurological recovery. This drop indicates successful reperfusion and improved cerebral autoregulation, due to a reduced final ischemic core.^16^ Large vessel occlusion itself can also influence the autoregulatory processes of the cerebral vessels,^17^ leading to changes of cerebral blood flow and subsequent clinical deterioration.^14,18^

We aimed to investigate the influence of flow restoration on the periprocedural blood pressure levels. We hypothesize that successful angiographic reperfusion of a large vessel occlusion is associated with an immediate periinterventional decrease of systemic blood pressure during mechanical thrombectomy.

## Methods

This is a retrospective, single-center analysis of consecutive patients with large vessel occlusion in the anterior circulation undergoing mechanical thrombectomy.

### Patient selection and data acquisition

Patients undergoing endovascular treatment in the anterior circulation with large vessel occlusion (intracranial internal carotid artery, M1, and proximal dominant M2 occlusions) between June 2016 and January 2018 were included in the study. Data were retrospectively extracted from an institutional review board-approved database and clinical charts. Neuroradiological procedural data acquisition was performed by treating physicians and entered in a standardized form immediately after the endovascular therapy procedure. Throughout the procedure, blood pressure was monitored either at 1-min intervals via an intra-arterial catheter or at 5-min intervals using a noninvasive blood pressure cuff. Subsequently, electronic blood pressure data were extracted from the patient’s anesthesia reports.

Patients meeting the following inclusion criteria were selected: (1) age > 18 years, (2) treatment performed under local anesthesia or conscious sedation (3) complete periprocedural and data sets available.

### Data analysis

Data assessment included patients’ age, admission National Institutes of Health Stroke Scale (NIHSS) score, modified Rankin Scale (mRS) score before admission, time between symptom onset/last seen well, groin puncture and flow restoration and administration of intravenous thrombolysis.

Anesthesia protocols were analyzed and systolic, diastolic and mean arterial pressure values throughout the procedure were assessed, including periprocedural minimal, maximal, initial and endpoint blood pressure values, as well as values before and after the thrombectomy maneuver. Application of circulation effective medication (propofol, opioids, sympatholytic antihypertensive medication) was determined by the treating anesthesiologist. The most common medication protocol included: continuous infusion of Remifentanil (0.02–0.05 μg/kg/min), continuous infusion of Propofol (1.5–2 μg/kg/h) or bolus application of Sufentanil 5 μg for conscious sedation; bolus application of Ebrantil/Urapidil i.v. (10 mg) and continuous infusion of Noradrenalin as antihypertensive or vasopressor medication.

The last attempt of thrombectomy maneuver resulting in successful angiographic reperfusion or interruption of the procedure was evaluated, and systemic blood pressure values 5 min before and 5 min after the maneuver were compared as primary outcome. Successful reperfusion was defined as a modified Thrombolysis in Cerebral Infarction score ⩾ 2b.^19^

### Statistical analysis

Statistical analyses were conducted using R software version 4.2.2. Patients were categorized into two groups based on angiographic reperfusion success. Univariate associations were evaluated using the Mann–Whitney test (non-normally distributed data) and unpaired *t*-test (normally distributed data). Logistic regression analysis was performed using the difference in systolic blood pressure 5 min before and 5 min after the last maneuver as the dependent variable, and all variables that showed significant differences in univariate comparisons were included as independent variables. A significance threshold of *p* < 0.05 was applied. For patients who did not receive any circulation-effective medication, blood pressure values were compared before and after recanalization using the Welch two-sample *t*-test.

## Results

Among a total of 253 patients identified, 85 (34%) procedures were performed under general anesthesia and in 34 (13%) cases no complete periprocedural data sets were available. 134 (53%) met the inclusion criteria and were included in the analysis. Successful angiographic reperfusion was achieved in 117 patients (87%).

As shown in Table 1, analysis of baseline variables, clinical and periprocedural characteristics displayed a significant reduction of systolic blood pressure (SBP) in patients with successful reperfusion 5 min after recanalization compared to those with unsuccessful reperfusion (10.2 ± 14.6 vs 3.24 ± 8.65 mm Hg, *p* = 0.009). No statistically significant differences in systolic blood pressure reduction were observed between the patients with TICI 2b (*n* = 58) and TICI 3 (*n* = 59) angiographic reperfusion: (5 (0–15) vs 5 (0–15) mm Hg, *p* = 0.923).

**Table 1.** Baseline variables, clinical and periprocedural characteristics for the group of patients with versus without successful reperfusion.

Variable	Successful reperfusion, *N* = 117 (87%)	Unsuccessful reperfusion, *N* = 17 (11%)	*p*-Value
Age, mean (SD)	71.8 (12.0)	71.9 (9.97)	0.957
Women, *n* (%)	59 (50.4%)	8 (47.1%)	1.000
Comorbidities			
Arterial hypertension, *n* (%)	81 (69.2%)	11 (64.7%)	0.923
Diabetes, *n* (%)	26 (22.2%)	3 (17.6%)	1.000
Dyslipidemia, *n* (%)	17 (14.5%)	2 (11.8%)	1.000
Atrial fibrillation, *n* (%)	47 (40.2%)	6 (35.3%)	0.905
Active smoking	15 (12.8%)	4 (23.5%)	0.264
ASPECTS, median (IQR)	8 (6–9)	8 (7–9)	0.984
Iv.thrombolysis, *n* (%)	72 (61.5%)	5 (29.4%)	0.025
NIHSS on admission, median (IQR)	15 (11–18)	13 (9–16)	0.324
Occlusion location			
Tandem, *n* (%)	12 (10.3%)	5 (29.4%)	0.043
ICA, *n* (%)	26 (22.2%)	3 (17.6%)	1.000
MCA-M1, *n* (%)	75 (64.1%)	12 (70.6%)	0.987
MCA-M2, *n* (%)	16 (13.7%)	2 (11.8%)	1.000
Number of passes, median (IQR)	2 (1–2)	2 (2–4)	0.016
Reperfusion (TICI)			
1, *n* (%)	0	4 (23.5%)	
2a, *n* (%)	0	13 (76.5%)	
2b, *n* (%)	58 (49.6%)	0	
3, *n* (%)	59 (50.4%)	0	
Symptom onset to groin, mean (SD), min	231 (88.6)	288 (234)	0.514
Symptom onset to reperfusion, mean (SD), min	276 (92.8)	375 (232)	0.273
Admission to groin, mean (SD), min	65.5 (22.1)	145 (340)	0.368
Groin to reperfusion, mean (SD), min	46.3 (28.2)	71.2 (33.6)	0.009
NIHSS after 24 h, median (IQR)	8 (4–16)	14 (11–19)	0.027
NIHSS at discharge, median (IQR)	5 (1–9)	10 (5–14)	0.023
mRS at 24 h, median (IQR)	4 (3–5)	5 (4–5)	0.009
mRs at discharge, median (IQR)	3 (2–5)	5 (3–5)	0.03
Systolic BP at the beginning, mean (SD), mm Hg	158 (27.0)	152 (21.9)	0.350
Diastolic BP at the beginning, mean (SD), mm Hg	82.3 (14.7)	80.0 (12.7)	0.501
MAP at the beginning, mean (SD), mm Hg	108 (17.0)	104 (14.7)	0.390
Minimal systolic BP, mean (SD), mm Hg	131 (17.6)	134 (19.1)	0.628
Minimal diastolic BP, mean (SD), mm Hg	65.7 (10.9)	69.4 (11.4)	0.221
Minimal MAP, mean (SD), mm Hg	87.6 (11.6)	90.9 (12.9)	0.333
Maximal systolic BP, mean (SD), mm Hg	173 (22.8)	168 (18.2)	0.328
Maximal diastolic BP, mean (SD), mm Hg	90.4 (15.9)	90.6 (10.9)	0.947
Systolic BP mid-procedure, mean (SD), mm Hg	155 (19.0)	152 (26.0)	0.701
Diastolic BP mid-procedure, mean (SD), mm Hg	77.8 (12.3)	79.4 (12.6)	0.622
Systolic BP 5 min before last attempt, mean (SD), mm Hg	158 (19.9)	156 (19.7)	0.727
Diastolic BP 5 min before last attempt, mean (SD), mm Hg	79.0 (13.4)	80.3 (11.2)	0.674
Systolic BP 5 min after last attempt, mean (SD), mm Hg	147 (18.5)	153 (22.8)	0.383
Diastolic BP 5 min after last attempt, mean (SD), mm Hg	77.8 (16.5)	80.3 (12.7)	0.470
Systolic BP at the end, mean (SD), mm Hg	144 (16.2)	149 (19.0)	0.280
Diastolic BP at the end, mean (SD), mm Hg	75.0 (11.8)	82.9 (9.69)	0.005
Value of systolic BP difference 5 min before versus after reperfusion, mean (SD), mm Hg	10.2 (14.6)	3.24 (8.65)	0.009
Value of diastolic BP difference 5 min before versus after reperfusion, mean (SD), mm Hg	1.24 (14.0)	0.00 (5.59)	0.511
Number of patients with a positive systolic BP difference 5 min before versus after reperfusion, *n* (%)	81 (69.2%)	7 (41.2%)	0.045

ASPECTS: Alberta Stroke Programme Early CT Score; BP: blood pressure; ICA: internal carotid artery; Iv.thrombolysis: intravenous thrombolysis; IQR: interquartile range; MCA: middle cerebral artery; MAP: mean arterial pressure; NIHSS: National Institutes of Health Stroke Scale; mRS: Modified Rankin Scale; SD: standard deviation; TICI: thrombolysis in cerebral infarction.

Patients with successful reperfusion were more likely to receive IVT compared to those with unsuccessful reperfusion (61.5% vs 29.4%, *p* = 0.025). Tandem occlusion was observed more frequently in patients with unsuccessful reperfusion (10.3% vs 29.4%, *p* = 0.043). The time from groin puncture to reperfusion was significantly longer in patients with unsuccessful reperfusion (46.3 (28.2) vs 71.2 (33.6) min, *p* = 0.009).

Patients with successful reperfusion had significantly lower NIHSS scores after 24 h (8 (4–16) vs 14 (11–19), *p* = 0.027) and at discharge (5 (1–9) vs 10 (5–14), *p* = 0.023) compared to those with unsuccessful reperfusion. Additionally, the modified Rankin Scale (mRS) scores at 24 h and discharge were significantly lower in patients with successful reperfusion (4 (3–5) vs 5 (4–5), *p* = 0.009 and 3 (2–5) vs 5 (3–5), *p* = 0.03).

Logistic regression was performed using the presence of a positive systolic blood pressure difference 5 min before versus 5 min after last thrombectomy attempt as dependent variable. Successful reperfusion, the presence of tandem occlusion, the time from groin puncture to reperfusion and iv-thrombolysis were included as independent variables. Successful reperfusion emerged as an independent predictor for a positive change in systolic blood pressure, with an adjusted odds ratio of 1.34 (95% CI: 1.03–1.73, *p* = 0.0299; Table 2).

**Table 2.** Logistic regression analysis results for predictors of systolic blood pressure reduction 5 min after successful reperfusion.

Variable	Odds ratio	95% CI	*p*-Value
Tandem occlusion	1.13	0.88–1.44	0.8155
Successful reperfusion	1.34	1.03–1.73	0.0299
Groin to reperfusion	1.00	1.00	0.9372
Iv.thrombolysis	1.04	0.88–1.23	0.6767

In 66 patients (49%) the intervention was performed under local anesthesia and they did not receive any circulation effective medication. Table 3 describes blood pressure values 5 min before and 5 min after the last thrombectomy maneuver in these patients. A significant reduction of the systolic blood pressure was observed only in the successful reperfusion group (155 mm Hg ± 17 mm Hg vs 148 mm Hg ± 17 mm Hg; *p* < 0.001).

**Table 3.** Blood pressure values 5 min before and 5 min after the thrombectomy maneuver in cases without periinterventional application of circulation effective medication.

Variable	Successful reperfusion, *N* = 59 (89%)			Unsuccessful reperfusion, *N* = 7 (11%)		
	Before	After	*p*-Value	Before	After	*p*-Value
Systolic BP, mean (SD), mm Hg	155 (17)	148 (17)	<0.001	166 (20)	164 (23)	0.6036
Diastolic, mean (SD), mm Hg	78 (12)	78 (19)	0.6801	86 (15)	86 (16)	0.6036
MAP, mean (SD), mm Hg	103 (12)	101 (17)	0.2229	112 (16)	112 (18)	0.9962

## Discussion

In this retrospective study, we determined whether flow restoration during MT is associated with changes in systemic blood pressure in patients with acute ischemic stroke due to large vessel occlusion. We observed an immediate decrease of systolic blood pressure after flow restoration during MT under local anesthesia or conscious sedation. This suggests a complex interplay between endovascular stroke therapy and cardiovascular hemodynamics.

Several authors have delineated the progression and control of blood pressure, along with its impact on clinical outcomes during the initial hours following stroke treatment. The significance of blood pressure monitoring extends beyond the interventional procedure, impacting pre- and postoperative management and providing potential prognostic indicators for neurological outcomes.^20–24^ While the authors describe blood pressure changes hours and days after thrombectomy, our data show that this effect occurs immediately after vessel reperfusion. To the best of our knowledge, there has been no published data on the immediate decline of systolic blood pressure during an intervention conducted under conscious sedation or local anesthesia, with the exclusion of any influence on cardiovascular parameters attributable to the medication employed in general anesthesia.

In our assessment, we utilized blood pressure readings taken 5 min prior to the stent retrieval maneuver and 5 min thereafter. This approach accounts for the potential pain associated with for example, the stent retrieval maneuver itself, which may induce a brief elevation of blood pressure for the patient.

Our results also resonate with published data on blood pressure course and patterns of post-procedural blood pressure changes after endovascular stroke treatment. Mattle et al. showed that the course of elevated systolic blood pressure following acute ischemic stroke was observed to exhibit an inverse correlation with the extent of vessel recanalization. Prolonged elevation of systolic blood pressure was noted in cases of recanalization failure compared to instances where reperfusion was successful. As in our study, Mattle et al. observed no difference in diastolic blood pressure depending on the success of recanalization.^25^ The key distinction between this study and our results is that our study demonstrates the immediate effect of reperfusion on blood pressure. The observed blood pressure patterns in acute ischemic stroke before and after flow restoration suggest a complex cascade of physiological responses, involving the release of vasodilatory substances, alterations in autonomic tone, and the mechanical consequences inherent in the recanalization process as described previously.^26,27^

A thorough exploration of this relationship is essential for optimizing procedural safety, anticipating complications, and possibly tailoring individualized treatment plans. The significance of blood pressure management before and during endovascular therapy for acute ischemic stroke becomes evident when considering the findings of various studies. Hendén et al. emphasized the role of intraprocedural hypotension as an independent predictor for unfavorable neurological outcomes in patients undergoing endovascular therapy in general anesthesia.^11^ Similarly, Whalin et al. highlighted a 10% drop in mean arterial pressure from baseline as a significant risk factor for poor outcomes following thrombectomy under sedation.^28^ Collateral status is known to have a great impact on clinical outcome in stroke patients (as it influences the infarct core size and the final stroke volume) and is also a subject of influence of blood pressure dynamics during endovascular treatment.^29,30^ As Maïer et al. demonstrated, hypotension during EVT was linked to poorer outcomes specifically in patients with inadequate collateral status.^31^ Based on these findings, lowering blood pressure in acute stroke may seem counterintuitive, also in the pre-hospital phase.^15^ After successful reperfusion on the contrary, lower SBP targets could be an important strategy in optimizing clinical outcomes, as targeting SBP goals of less than 140 mm Hg and less than 160 mm Hg appear to be associated with improved clinical outcomes compared to an SBP target of less than 180 mm Hg.^23^

Building on these insights, it becomes crucial to integrate the published data with our own results. Specifically, attention should be directed toward anticipating and tolerating systolic and mean blood pressure drops after successful flow restoration to a certain extent as it could be an expected result after stent retrieval or aspiration maneuver. Moreover, further assessing the influence of the immediate periinterventional SBP reduction on clinical outcomes could add to the existing insights about the optimal management of blood pressure during endovascular treatment.

## Limitations

The retrospective nature of our study and the modest sample size necessitate caution in generalizing our results. This small sample size may not provide a comprehensive representation of the broader patient population experiencing failed reperfusion.

Moreover, the small number of cases limits our ability to perform detailed subgroup analyses.

Further, the absence of a control group undergoing a distinct anesthetic modality introduces an element of uncertainty regarding the exclusive attribution of blood pressure changes to the reperfusion procedure. This limitation highlights the necessity for further studies with larger sample sizes to validate our findings and provide more robust evidence.

However, the inclusion of detailed patient demographics, procedural characteristics, and anesthesia medication protocols enhances the internal validity of our findings.

## Conclusion

For patients undergoing mechanical recanalization under local anesthesia or conscious sedation, flow restoration was associated with an immediate significant reduction of systolic blood pressure values. These findings may provide valuable insights into the complex interplay between endovascular therapy and cardiovascular dynamics.
